# Supplementary material for: Unsupervised Machine Learning to Identify Patient Clusters and Tailor Perioperative Care in Colorectal Surgery
Source: Diagnostics (Basel). 2025 Aug 22;15(17):2124. doi: 10.3390/diagnostics15172124 (PMC12428037; doi:10.3390/diagnostics15172124)
Supplement: Supplementary file 1 [file diagnostics-15-02124-s001.zip › diagnostics-3726920-supplementary.pdf]

Supplementary files

Table S1 : List of available demographic, peri- and postoperative variables

Categorical variables (chi-square test, ** p<0.01)

Demographic Variables (test between 3 clusters)
Gender
Preoperative nutritional status assessment**
Smoker
Alcohol usage**
Diabetes mellitus
Severe heart disease**
Severe pulmonary disease**
Preoperative WHO performance score**
Recent immunosuppressive treatment
Preoperative chemotherapy**
Any radiotherapy to operating field**
Previous surgery to same abdominal region
Main procedure name**
Additional major procedures**
Procedure group**
Surgical approach**
Surgical approach group**
Operation converted**
New stoma**
Stomal Procedure**
Bowel anastomosis**
Type of bowel anastomosis**
Anastomotic technique**
Peritoneal soiling / contamination**
ASA physical status class**
General anaesthesia**

Perioperative Variables (test between 2 clusters)
Preoperative nutritional treatment**
Preadmission patient education given
Preoperative oral carbohydrate treatment
Oral bowel preparation**
Antibiotic prophylaxis before incision**
Thrombosis prophylaxis
Resection-site drainage**
Urinary drainage postop**
Previous PONV or motion sickness
PONV prophylaxis administered
Epidural or spinal anaesthesia**
Upper-body forced-air heating cover used
Forced-air heating cover used
Heated IV fluids used
Use of 0.9% NaCl**
Fluid administration guidance**
Nasogastric tube used postoperatively**
Energy Intake On day of surgery, postoperatively**

Energy Intake on Postoperative Day 1**<br>Mobilisation at all on day of surgery**<br>Mobilisation at all on POD1**<br>Mobilisation on postoperative day 1**<br>Mobilisation on postoperative day 2**<br>Mobilisation on postoperative day 3**<br>30 day follow up performed**<br>Stimulation of gut motility**<br>Postoperative epidural analgesia**<br>Patient Weight On postoperative day 1**<br>Patient Weight on POD1**<br>Weight change on POD1**<br>Balanced fluids day 0**<br>Postop oral energy intake POD0**<br>Postop oral energy intake POD1**

Postoperative Variables (test between 3 clusters)	
Postoperative epidural analgesia**	Other infectious complication**
Postoperative use of NSAIDS**	Cardiovascular complications**
Nasogastric tube reinserted**	Heart failure**
Observed nausea, retching and vomiting - On day of surgery**	Acute myocardial infarction**
Observed nausea, retching and vomiting - On postoperative day 1**	Deep venous thrombosis**
Observed nausea, retching and vomiting - On postoperative day 2**	Pulmonary embolus**
Observed nausea, retching and vomiting - On postoperative day 3**	Cerebrovascular lesion**
Opioid use - On day of surgery**	Cardiac arrhythmia**
Opioid use - On postoperative day 1**	Cardiac arrest**
Opioid use - On postoperative day 2**	Other cardiovascular complication**
Opioid use - On postoperative day 3**	Renal, hepatic, pancreatic and gastrointestinal complications**
Discharged within 30 postop days**	Renal dysfunction**
Discharged to**	Urinary retention**
Complications at all during primary stay**	Hepatic dysfunction**
Respiratory complications**	Pancreatitis
Lobar atelectasis**	Gastrointestinal haemorrhage**
Pneumonia**	Nausea or vomiting**
Pleural fluid**	Obstipation or diarrhoea**
Respiratory failure**	Other organ dysfunction**
Pneumothorax	Surgical complications**
Other respiratory complication**	Anastomotic leak**
Infectious complications**	Urinary tract injury**
Wound infection**	Mechanical bowel obstruction**
Urinary tract infection**	Postoperative paralytic ileus**
Intraperitoneal or retroperitoneal abscess**	Deep wound dehiscence**
Sepsis**	Intraoperative excessive haemorrhage**
Septic shock**	Postoperative excessive haemorrhage**
Infected graft or prosthesis**	Other surgical technical complication or injury
Aspiration of gastric contents	Complication(s) related to epidural or spinal anaesthesia**
Hypotension	Epidural hematoma or abscess
Hypoxia	Other EDA or spinal related complication**
Prolonged postoperative sedation**	Anaesthetic complications
	Psychiatric complications**
	Asthenia or tiredness**

Other anaesthetic complication(s)^^	Pain**
WHO Performance Score at 30 days** postoperatively**	Injuries
Complications at all after primary stay**	30 day survival**

Table S2 : Detailed analysis of postoperative variables (beyond the predefined top five recovery goals and the top 10 clinical outcome items) :

Demographic Variable (Numeric)	Cluster 1 (n = 490)	Cluster 2 (n = 157)	Cluster 3 (n = 734)	P-value	Sample mean (n=1381)
Age	61.17	63.21	60.80	0.24	61.20
Weight 6 months prior to admission	73.98	74.89	75.03	0.36	74.64
Preoperative body weight	73.26	73.18	74.35	0.46	73.83
Preoperative weight change	-1.05	-1.25	-1.08	0.85	-1.09
Height	168.72	168.20	169.15	0.46	168.89
BMI	25.64	25.80	25.95	0.58	25.8
Length of incision	13.06	13.12	12.73	0.72	12.89

Demographic Variable (Categorical)	Cluster 1 (n = 490)	Cluster 2 (n = 157)	Cluster 3 (n = 734)	P-value
Gender	57.9% male	50.9% male	58.0% male	0.24
Smoker	79.3% no<br>2.0% stopped	75.1% no<br>1.3% stopped	73.7% no<br>1.5% stopped	0.13
Alcohol usage	53.4% no<br>0.4% stopped	77.8% no<br>0.6% stopped	83.6% no<br>0.3% stopped	<0.001
Diabetes mellitus	89.6% no<br>8.9% medication<br>1.5% diet control	84.1% no<br>15.3% medication<br>0.6% diet control	87.5% no<br>11.8% medication<br>0.7% diet control	0.13
Severe heart disease*	2.0% no<br>2.0% yes	61.1% no<br>6.4% yes	90.4% no<br>9.6% yes	<0.001
Severe pulmonary disease*	0% no<br>4.1% yes	64.3% no<br>31.8% yes	95.9% no<br>4.1% yes	<0.001

Perioperative Variable	Cluster 1 (n = 370)	Cluster 2 (n = 1011)	**P-value**
Core body temperature at end of operation	36.40	36.27	<0.0001
IV volume of crystalloids intraoperatively	2517.89	1307.12	<0.0001
IV volume of colloids intraoperatively	395.13	81.89	<0.0001
Total IV volume of fluids intraoperatively	2980.91	1376.44	<0.0001
Total IV volume of fluids day zero	4227.59	2155.41	<0.0001
Morning weight<br>- On postoperative day 1	78.79	74.01	<0.0001
Weight change day 1	2.09	0.80	<0.0001
Morning weight<br>- On postoperative day 2	80.44	73.09	<0.0001
Weight change day 2	2.71	0.83	<0.0001
Morning weight<br>- On postoperative day 3	80.49	72.61	<0.0001
Weight change day 3	2.53	0.53	<0.0001
Oral fluids, total volume taken<br>- On day of surgery, postoperatively	658.57	1009.79	<0.0001
Oral fluids, total volume taken<br>- On postoperative day 1	1350.56	1617.10	<0.0001
Oral fluids, total volume taken<br>- On postoperative day 2	1350.18	1598.84	<0.0001
Oral nutritional supplements, energy intake<br>- On day of surgery, postoperatively	52.70	128.79	<0.0001

Oral nutritional supplements,<br>energy intake - On postoperative day 1	194.60	349.12	<0.0001
Oral nutritional supplements,<br>energy intake - On postoperative day 2	207.97	314.14	<0.0001
Oral nutritional supplements,<br>energy intake - On postoperative day 3	120.16	177.25	0.0001

Postoperative Variable	Cluster 1<br>(n = 535)	Cluster 2<br>(n = 667)	Cluster 3<br>(n = 162)	**P-value**
Total length of stay (nights)	8.97	5.96	20.08	<0.0001
Time to passage of flatus (nights)	1.91	1.61	3.03	<0.0001
Time to passage of stool (nights)	2.66	2.12	3.84	<0.0001
Time to tolerating solid food (nights)	2.46	2.07	6.93	<0.0001
Time to termination of urinary drainage (nights)	2.05	1.81	3.88	<0.0001
Time to pain control with oral analgesics (nights)	2.64	1.81	5.43	<0.0001
Patient-reported maximum pain (VAS)<br>- On day of surgery	3.13	3.22	3.23	0.82
Patient-reported maximum pain (VAS)<br>- On postoperative day 1	3.68	3.74	4.50	0.0006
Patient-reported maximum pain (VAS)<br>- On postoperative day 2	2.96	2.682	3.82	<0.0001
Patient-reported maximum pain (VAS)<br>- On postoperative day 3	2.30	1.92	2.93	<0.0001
Patient-reported maximum nausea (VAS)<br>- On day of surgery	0.89	0.43	0.18	<0.0001
Patient-reported maximum nausea (VAS)<br>- On postoperative day 1	1.43	0.51	0.98	<0.0001
Patient-reported maximum nausea (VAS)<br>- On postoperative day 2	1.22	0.31	1.16	<0.0001
Patient-reported maximum nausea (VAS)<br>- On postoperative day 3	1.03	0.17	1.34	<0.0001
Length of stay<br>(nights in hospital after primary operation)	8.27	5.22	19.06	<0.0001
Time between operation and follow up (nights)	42.89	41.66	47.95	<0.0001

* The leftover percentage presents patient status of 'unknown'

Clustering results for perioperative variables. BMI – Body Mass index; IV—intravenous; VAS – Visual Analog Scale

Table S3 : Hyperparameter selection (Silhouette score comparison between number of clusters)

Hyperparameter (number of clusters) selection on K-means method.

(a) Hyperparameter selection and the clustering performance on Demographic and Perioperative variables.

**Demographic Variables**	**Silhouette Score**
Number of clusters = 2	0.120
Number of clusters = 3	0.164
Number of clusters = 4	0.163
Number of clusters = 5	0.162
**Perioperative Variables**	**Silhouette Score**
Number of clusters = 2	0.141
Number of clusters = 3	0.105
Number of clusters = 4	0.099
Number of clusters = 5	0.071

(b) Hyperparameter selection and the clustering performance on combined Demographic and Perioperative variables.

**Demographic + Perioperative Variables**	**Silhouette Score**
Number of clusters = 2	0.137
Number of clusters = 3	0.111
Number of clusters = 4	0.090
Number of clusters = 5	0.058

(c) Hyperparameter selection and the clustering performance on the Outcome variables.

**Outcome Variables**	**Silhouette Score**
Number of clusters = 2	0.341
Number of clusters = 3	0.362
Number of clusters = 4	0.277
Number of clusters = 5	0.174

Table S4 : Missing variables

Demographic variables	MissingDataPercentage
Preoperative weight change	40.48
Weight 6 months prior to admission	40.41
Severe pulmonary disease	39.03
Severe heart disease	39.03
Length of incision	33.96
* Alcohol usage	16.44
BMI	0.22
Height	0.22
* Gender	0
Diabetes mellitus	0
* Smoker	0
Preoperative body weight	0
Age	0

Perioperative Variables	MissingDataPercentage
* Morning weight - On postoperative day 1	34.9
Weight change on POD1	34.54
Oral fluids, total volume taken - On postoperative day 2	34.03
Weight change day 3	31.57
Morning weight - On postoperative day 3	31.57
Oral fluids, total volume taken - On postoperative day 1	29.11
Weight change day 2	27.81
Oral fluids, total volume taken - On day of surgery, postoperatively	27.73
Morning weight - On postoperative day 2	27.59
Oral nutritional supplements, energy intake - On postoperative day 3	25.63
Oral nutritional supplements, energy intake - On postoperative day 2	21.07
* Oral nutritional supplements, energy intake - On postoperative day 1	17.81
* Oral nutritional supplements, energy intake - On day of surgery, postoperatively	16.65
Core body temperature at end of operation	3.84
IV volume of colloids intraoperatively	0.8
IV volume of crystalloids intraoperatively	0.8
* Total IV volume of fluids day zero	0
Total IV volume of fluids intraoperatively	0

Recovery goals	MissingDataPercentage
Weight change day 2	27.81
Time to tolerating solid food (nights)	26.14
Mobilisation on postoperative day 2	26
* Total IV volume of fluids day zero	0
Weight change > 2.5kg POD2	27.81

Clinical Outcomes	MissingDataPercentage
Re-operation(s)	59.52
Nasogastric tube reinserted	39.1
Patient-reported maximum pain (VAS) - On postoperative day 1	2.46
Respiratory complications	0
Infectious complications	0
Renal dysfunction	0
Anastomotic leak	0
Postoperative paralytic ileus	0
Date of death	0

BMI – Body Mass index; IV—intravenous; POD – postoperative day

Figure S1 : Preprocessing data scheme

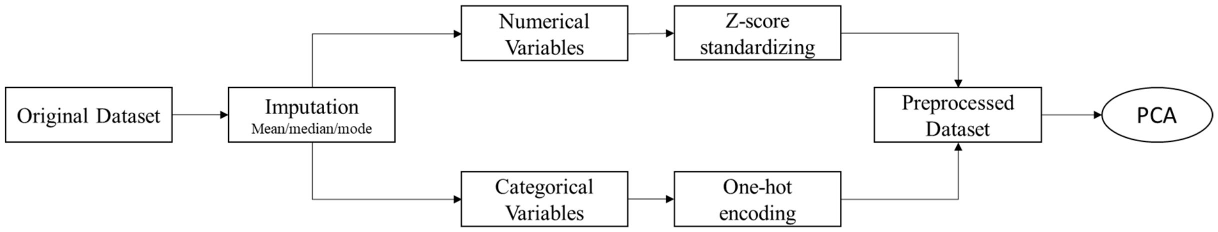

The data preprocessing pathways before performing K-means clustering.

Figure S2 : Schematic representation of clustering approach

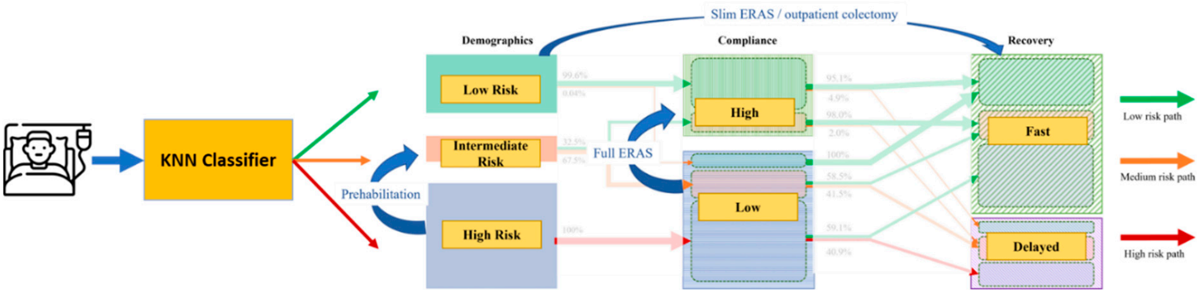

To translate our clustering results into a predictive tool for clinical use, we developed a supervised classification model. The risk groups identified via K-means clustering were used as the outcome labels for training a K-Nearest Neighbors (KNN) classifier. The purpose of this model is to prospectively classify new patients into these established risk strata, thereby enabling the implementation of risk-stratified treatment pathways.
